# EDUCATION ON COMMUNICATION WHILE WEARING FACEMASKS USEFUL DESPITE THE END OF COVID-19 EMERGENCY

**DOI:** 10.1093/geroni/igae098.0513

**Published:** 2024-12-31

**Authors:** Clarissa Shaw, Kristine Williams, Carissa Coleman, Lynn Nakad

**Affiliations:** University of Iowa, Iowa City, Iowa, United States; University of Kansas Medical Center, Kansas City, Kansas, United States; University of Kansas Medical Center, Kansas City, Kansas, United States; University of Iowa, Iowa City, Iowa, United States

## Abstract

Communication with nursing home residents by formal caregivers is complicated with the use of facemasks. Yet facemask use is necessary to limit the spread of disease during surges of communicable respiratory illnesses. The purpose of this study was to develop and pilot test a web-based educational module to promote successful communication with nursing home residents while wearing facemasks. To create CHATO-PPE, we completed a scoping review of 39 articles and interviewed 19 formal caregivers, nursing residents, and family members from four states on best practices for communication with facemasks. We integrated these results to create a 30-minute web-based module. The module includes three videos we produced with a scenario that begins as an undesirable care encounter and with our education improves with practical communication tips and then becomes a successful care encounter with the integration of person-centered communication. To evaluate our module, we recruited a convenience sample of 26 formal caregivers in nursing homes. Confidence in dementia care measured by the CODE scale increased significantly from before to after the modules (p=.009). Greater than 90% of participants agreed that they would recommend the course to others, use the training in their job, and were satisfied overall. In post-education interviews, learners stated that mask use is still prevalent in nursing homes during surges of respiratory illnesses and CHATO-PPE provides helpful and feasible tips to implement during these times while also serving as a reminder of communication best-practices that can be used regardless of facemask use.

